# Chiropractic’s role in strengthening rehabilitation in health systems

**DOI:** 10.1186/s12998-026-00654-y

**Published:** 2026-06-14

**Authors:** Katie de Luca, Afua Adjei-Kwayisi, Nora Bakaa, Christoffer Andre Børsheim, Amy Bowzaylo, Richard Brown, Raul Carrillo, Patricia Estrada, Jean-Luc Gauthier, Jordan A. Gliedt, Brett Guist, Neil Langridge, Peter Tuchin, Michael Vianin, Arnold Y. L. Wong

**Affiliations:** 1 Chiropractic, School of Health, Medical and Applied Sciences, CQUniversity, Brisbane, QLD 4001 Australia; 2 Physical Therapy and Rehabilitation Department, Greater Accra Regional Hospital, Accra, Ghana; 3 Institute of Disability and Rehabilitation Research, OntarioTech University, Oshawa, Canada; 4 Faculty of Medicine, University of Bergen, Bergen, Norway; 5 Department of Physical Medicine and Rehabilitation, Royal Military Medical Services, Janabiayh, Kingdom of Bahrain; 6 AECC School of Chiropractic, Health Sciences University, Bournemouth, UK; 7 Rebioger Clinics, Monterrey, Mexico; 8 Logan University Health Center, Saint Louis, USA; 9 Département de Chiropratique, Université du Québec à Trois-Rivières, Trois-Rivières, Canada; 10 Department of Neurosurgery, Medical College of Wisconsin, Milwaukee, WI USA; 11 Canadian Memorial Chiropractic College, Toronto, Canada; 12 Health Sciences University, Bournemouth, UK; 13 Sydney, Australia; 14 Unité de Chiropratique, Centre Hospitalier Universitaire Vaudois, Lausanne, Switzerland; 15 Department of Rehabilitation Sciences, The Hong Kong Polytechnic University, Kowloon, Hong Kong SAR China

**Keywords:** Rehabilitation, Chiropractic, Health systems, Health system research, Disability, Education, Healthcare

## Abstract

**Background:**

Rehabilitation is described as the care needed when a person is experiencing, or is likely to experience, limitations in everyday functioning. In 2019, it was estimated that 2.4 billion people were living with a condition that could benefit from rehabilitation. Chiropractors have contributed meaningfully to the delivery of rehabilitation due to their training in musculoskeletal health and person-centred care. Building on the World Federation of Chiropractic’s chiropractic rehabilitation competency framework, this commentary highlights chiropractic's role in strengthening rehabilitation in health systems. Under the “Role of Chiropractic in Rehabilitation”, we explore the postoperative setting, sports injury recovery, and neurorehabilitation. We share how the “Integration of Chiropractors into Health Systems” exists in multidisciplinary rehabilitation teams, publicly funded health systems and in hospitals. We discuss the “Economic and Social Impacts” of the chiropractic profession in low- and middle-income countries, for equity, diversity and inclusion, and regarding cultural competencies. We present “Challenges for the Chiropractic Profession” that include varying legislations within and between countries, limited recognition among other healthcare professionals, and advanced integration within multidisciplinary rehabilitation programs. Finally, we make a “Call to Action” for theory driven implementation strategies, to advocate for improved recognition of the chiropractic profession, and to enhance interprofessional education and advance rehabilitation in chiropractic curricular.

**Conclusions:**

Chiropractors can play an important role in strengthening rehabilitation in health systems as they deliver interventions for rehabilitation for people with health conditions throughout the life course, and across the continuum of care. However, barriers remain. As a profession we must align with global rehabilitation priorities and uptake taxonomies to enable equitable access to chiropractors focused on optimising function and participation.

## Background

Rehabilitation is the care needed when a person experiences or is likely to experience limitations in everyday functioning due to aging or a health condition, including chronic diseases or disorders, injuries, or trauma [1]. It is defined by the World Health Organization (WHO) as “a set of interventions designed to optimise functioning and reduce disability in individuals with health conditions in interaction with their environment.” In 2019, it was estimated that 2.4 billion people were living with a condition that could benefit from rehabilitation. This number is expected to grow due to factors such as increasing longevity, an increased number of people living with chronic diseases, and disability [2]. Disability arises from the interaction between individuals with a health condition and various personal and environmental factors [3].

WHO has developed a framework to help conceptualise the interconnectivity between health and disability. This framework, called the International Classification for Functioning and Disability (ICF), is a biopsychosocial framework that conceptualises health and disability as dynamic interactions between a health condition, body functions and structures, activities, participation, and contextual factors [4]**.** It shifts focus towards overall functioning, supporting patient-centred, function-oriented care. The ICF enables comprehensive assessment, guides multimodal intervention planning, and facilitates interprofessional communication by providing a standardised language for understanding and addressing the full impact of musculoskeletal conditions. Within the ICF framework, rehabilitation is understood as a coordinated, goal-directed process aimed at optimising functioning and participation.

**Fig. 1:**
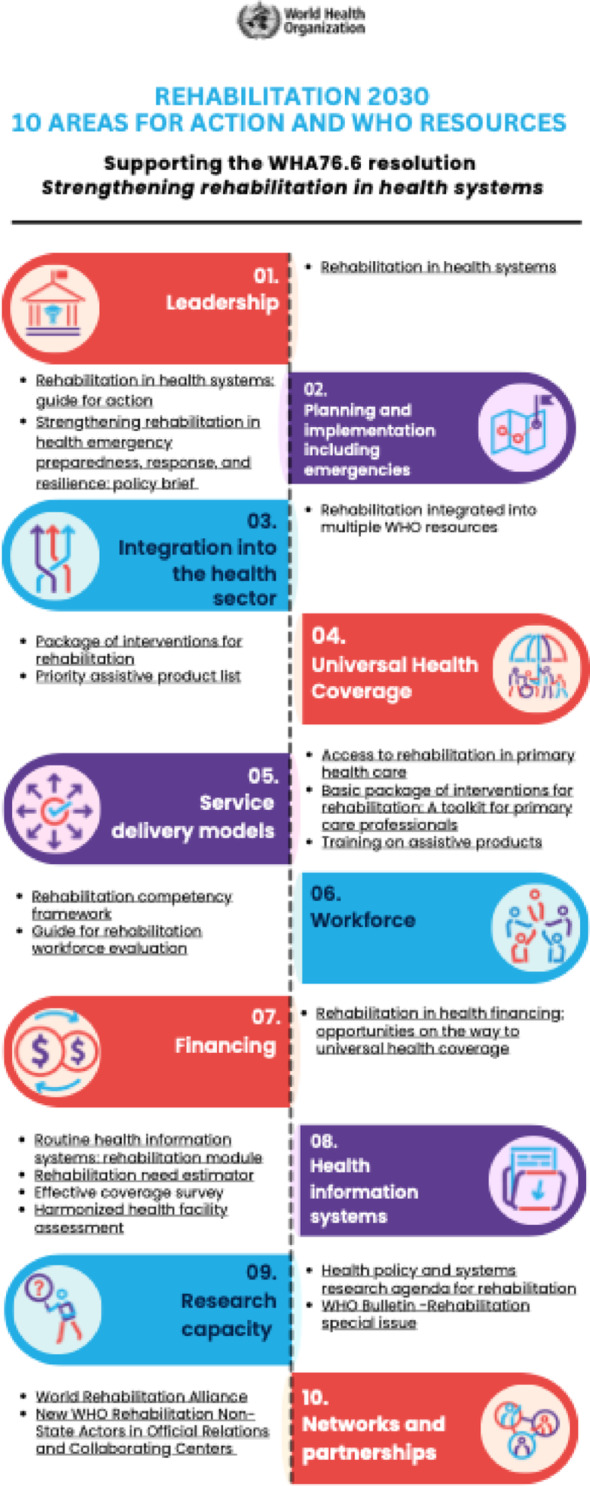
Rehabilitation 2030: 10 areas for action and who resources

Rehabilitation 2030 [5] draws attention to the profound unmet need for rehabilitation worldwide and highlights the importance of strengthening health systems to provide rehabilitation. Rehabilitation 2030 is a call to action, articulated into 10 priority areas (Fig. 1), to scale up rehabilitation so that countries can be prepared to address the evolving needs of diverse populations. It has been recommended that health systems include a multidisciplinary workforce to fully and effectively address the wide range of rehabilitation needs across these diverse populations [6].

In response to WHO’s call for non-governmental organisations, associations, and institutions to disseminate their rehabilitation-related competency frameworks, [4] the World Federation of Chiropractic (WFC) developed a chiropractic rehabilitation competency framework to support WHO's strategic plan for rehabilitation [7]. Building on this, this commentary highlights the role of chiropractic in the rehabilitation sector and advocates for strengthening of local, regional, and global multidisciplinary rehabilitation workforces.

### The role of chiropractic in rehabilitation

The rehabilitation workforce encompasses a wide range of health professions that deliver interventions for rehabilitation across different health system levels, and in settings such as hospitals, schools, private practice, workplaces, sports fields, and people’s homes. Despite the fact chiropractors are well-positioned, they remain under-utilised and poorly integrated within most health systems. A global scoping review of 328 studies found that the median 12-month utilisation of chiropractic services was only 9.1% of the population, with no meaningful increase over a 35-year period (1980–2015) [8], despite rising prevalence of chronic musculoskeletal disorders and disability worldwide.

Chiropractors possess a broad and relevant skill set, where they perform comprehensive musculoskeletal assessment and neurological and orthopaedic examination. They deliver interventions such as spinal and peripheral manipulation, mobilisation, exercise therapy, and patient and caregiver education, which are collectively recommended in managing musculoskeletal conditions [9–13]. Such interventions can optimise function and support individuals to participate fully in daily life, work, and community roles. Within healthcare systems, chiropractors are able to contribute across a continuum of care where their roles include but are not limited to, primary contact musculoskeletal providers, members of an interdisciplinary teams, patient educators and advocates, and referral gatekeepers [14, 15]**.** Further, by embracing the ICF framework, we believe chiropractors could be better accepted to address impairments, activity limitations, and participation restrictions, to address population-level rehabilitation needs.

Utilising evidence from rehabilitation competencies and frameworks, rather than treating rehabilitation as the delivery of an isolated intervention, will enable chiropractors to serve a variety of roles, depending on the health care needs of their patient. By optimising function and a person-centered approach, here, we present three examples of the delivery of interventions for rehabilitation by chiropractors in a postoperative setting, for sports injury recovery, and in neurorehabilitation.

1. *Patients recovering from spinal surgeries had enhanced patient functioning and recovery.* Many individuals with a history of spine surgery experience ongoing pain, functional deficit, or require reoperation [16, 17]. A retrospective cohort study revealed that adults with ongoing lumbosacral radiculopathy at least one year after lumbar discectomy who received spinal manipulative therapy, had a significantly lower rate of lumbar reoperation (7%) compared to matched controls receiving usual medical care (13%), and that this association persisted over two years’ follow-up [18]. Secondly, postoperative pain management guidelines following spine surgery recommend education and prescribed exercise tailored to surgical healing timelines to aid functional improvements and reduce medication use [19], and adjunctive management approaches include nonpharmacologic modalities like early mobilisation, cognitive behavioral therapy, and mindfulness-based interventions [20]. Favourably, recent evidence suggests that interventions provided by chiropractors including exercise and manual therapy were associated with favourable outcomes for individuals with prior spine surgery, including pain reduction, functional enhancement, and a reduced risk of reoperation [16–18, 21]. As such, chiropractors have the potential to reduce the risk of reoperation, support recovery and optimise function.
2. *Athletes seek treatment for sports-related injuries from chiropractors as part of a multidisciplinary environment to optimise recovery and improve performance.* Sports injuries are a common cause of pain and disability that can negatively impact an individual’s quality of life and well-being [22]. Worryingly, between 14 and 32% of competitive athletes are forced to retire because of a career ending injury [23]. Chiropractors can act as key providers of rehabilitation, as they actively participate in both preventive care and post-injury management [24]. Research in Australia has shown that chiropractors who ‘often’ treat athletes were more likely to report having more referral relationships with general practitioners, physiotherapists, podiatrists and/or medical specialists [25]. At the 2017 World Games*,* 16% of all athletes received chiropractic treatment [26] and two-thirds of Canadian national team athletes have reported using chiropractic care [27]. Evidence shows that interventions for rehabilitation delivered by chiropractors optimise movement and decrease recovery times [28] as well as maintain participation in athletic activity [29].
3. *Patients with neurological conditions may find improved functional outcomes.* While not curative, guideline concordant care includes holistic rehabilitation (fatigue management, physical activity, psychological interventions and chronic pain management) that can help alleviate chronic neurological disorders, through improvements in mobility and stability, and enhancing quality of life [30]. From an observational study of patients at a neurorehabilitation hospital, chiropractors managed patients with brain injury from trauma, haemorrhage, infarction, general anoxia, cervical spinal cord injury, ankylosing spondylitis, traumatic polyarthropathy, and respiratory failure with encephalopathy [31]. Chiropractors delivered interventions for rehabilitation that included myofascial therapies, percussion, muscle stretching, and thrust manipulation, with care adapted to participant limitations or conditions [31]. Manual therapies including mobilisation and manipulation, applied not as an isolated, standalone intervention but through the lense of rehabilitation can have positive effects on pain as well as the readiness to return to sport in patients with persistent post-concussive symptoms [32]. Although evidence supporting chiropractor’s involvement in neurorehabilitation remains limited, we believe chiropractors can optimise function in people with neurological conditions through the delivery of rehabilitation that includes exercise, vestibular rehabilitation, graded activity, and psychological support.

### Integration of chiropractors into health systems

Rehabilitation is an essential health service and crucial for achieving universal health coverage. Addressing challenges and leveraging opportunities to advance integration at the micro, mezzo and macro levels by intrinsic and extrinsic chiropractic stakeholders is needed. The Rehabilitation 2030 Initiative emphasises that efforts to strengthen rehabilitation should be directed towards supporting the health system as a whole and integrating rehabilitation into all levels of health care [6]. For chiropractors, barriers to integration occurs at many levels, particularly regarding regulatory complexities within and between countries and due to limited, heterogeneous evidence of cost-effectiveness within teams [33]. Here, we present three examples of how chiropractors have been integrated into primary care settings, publicly funded health systems and hospital outpatient rehabilitation departments.

1. *Collaborative environments where chiropractors work within multidisciplinary rehabilitation teams.* Mior et al., demonstrated that when chiropractors are embedded within primary-care teams, medical practitioners adjust their management of spine-related disorders in ways that improve care efficiency and patient outcomes [34]*.* Upon examining attitudes toward integrating chiropractors into primary care teams, there were generally positive attitudes between chiropractors and medical practitioners; however, the successful integration was based on factors such as communication, clear role definitions, and mutual trust [35]. Drawing from an interpretivist paradigm, Toloui-Wallace identified various physical (floor plans), social (identity and discourse) and organisational (appointment durations) factors that influenced the nature of collaborative environments between chiropractors, osteopaths and physiotherapists [36]. These factors were largely not fixed and likely responsive to change, suggesting progressive musculoskeletal healthcare should be less divisive and open to diverse clinicians practicing together. Practice characteristics within Switzerland show that while most chiropractors practice in a group setting with other chiropractors, a shift toward multidisciplinary settings is taking place [37]. Practical steps for chiropractors to participate in collaborative environments include engaging in case discussions and team rounds, providing timely and jargon free medical reports, building relationships through co-management, showcasing diagnostic skills and being reliable team players [38]. Collectively, multidisciplinary teams involving chiropractors have been shown to be feasible across diverse settings, when education, role clarity, patient preference, and access to care can be enhanced [39, 40].
2. *Overcoming variations and complexities for chiropractic in publicly funded health systems.* Chiropractors are integrated as part of public health systems in Norway, Sweden, Denmark and Switzerland, and conditionally available in Australia, New Zealand, the United Kingdom, the United States and Canada. As an example, the federally funded Veterans Affairs (VA) system in the United States reported a drastic increase in both unique patients and utilisation of chiropractic services until 2015 [41] when access and infrastructure greatly increased. Subsequently, the chiropractic profession was legislatively advanced in the VA in 2023 [42]. In the Canadian Forces Health Services, chiropractors were successfully integrated alongside physiotherapists, occupational therapists, and physicians when financial and resource barriers were addressed [43]. In public-funded primary care teams in Canada, Kopansky-Giles et al. advocated for maximising scope of practice to manage musculoskeletal cases [40]. Results showed that the provision of musculoskeletal care by chiropractors without economic barriers was desirable and highly valued by all members of the primary care team [40]. Furthermore, the emerging integration of chiropractic in publicly funded healthcare facilities has been associated with an overall higher level of satisfaction in patients suffering from spine pain [44]. As the rehabilitation workforce demand increases, there is a growing need for the chiropractic profession to update approaches beyond isolated and passive spinal manipulation. This will involve recognising, utilising and overcoming workforce specialism and equity concerns [5].
3. *Advocating for the delivery of rehabilitation by chiropractors in hospital departments.* The inclusion of chiropractors in hospital outpatient rehabilitation departments has progressed differently and varies greatly worldwide. Switzerland and Denmark are leading examples of the successful integration of chiropractic care into the hospital system [45, 46]. While in the United States, endeavours exist where chiropractic has been integrated into private hospitals over the last decade [47]. Examples of where chiropractors work with neurosurgeons and orthopaedic surgeons include providing prehabilitation for patients with symptomatic lumbar spinal stenosis [48]. Further collaborative opportunities exist for chiropractors due to challenges with postoperative recovery and rehabilitation and unmet needs for peer and emotional support [49]. In these settings, chiropractors may contribute to rehabilitation through musculoskeletal management and support for optimising function and participation. However, many challenges remain for integration of chiropractors within hospital-based settings, where healthcare systems in many countries are not structured optimally, and there is a lack of funding for health policy and systems research (HSPR) in integrating rehabilitation broadly [50]. In chiropractic, for example, professional legitimacy, scepticism, perceived service duplication, credentialing, administrative burdens and reimbursement are some of the barriers hospital systems must overcome for integration [51]. By improving interprofessional communication, developing policies to support the integration of chiropractors in hospital rehabilitation departments, and eliminating inefficiencies broadly across healthcare systems, [52] a more efficient management of musculoskeletal conditions and greater patient satisfaction is anticipated.

### Economic and social impacts

Integrating chiropractors in health systems to provide interventions for rehabilitation can likely lead to cost savings for healthcare systems due to reduced reliance on surgical interventions and pharmaceuticals, and increased access to patient centred care for neuromusculoskeletal conditions [53]. Additionally, improved patient outcomes and satisfaction contribute to overall societal well-being. Here, we present three examples of the impact that chiropractors may have when integrated into health systems in low- and middle-income countries (LMICs), for equity, diversity and inclusion (EDI) and regarding cultural competencies.

1. *Low- and middle-income countries.* In LMICs, more than 50% of people do not have access to the rehabilitation they require [5]. Various barriers, such as inadequate healthcare infrastructure, shortage of trained professionals and financial constraints, subsequently create rehabilitation inequities in these regions. Specifically, Gupta et al. [54] assessed the need for human resources for rehabilitation, indicating that LMICs had less availability of skilled health personnel, and that lack of transportation or inequality in the geographical distribution of services within a country hampers access to rehabilitation. We argue, since the economic backbone of most LMICs is manual labour, the provision of interventions for rehabilitation for prevalent conditions like back pain, neck pain and other musculoskeletal disorders by chiropractors is warranted. With caution however, there is limited evidence that shows an economic benefit of chiropractors of providing rehabilitation in LMIC’s. Rehabilitation itself, when community based and where tasks were shifted from more highly trained healthcare professionals to other health workers with less training and fewer qualifications (such as community health workers), has been shown to be economically valuable [55]. Where the greatest opportunity may lie for chiropractic is the provision of low-cost, conservative musculoskeletal care. Efforts to enhance access to rehabilitation in LMICs must include education of health policy makers, policy development, training local practitioners, and raising public awareness about disability.
2. *Advancing Equality, Diversity, and Inclusion in public health in relation to people with disabilities.* People with disabilities constitute approximately 1.4% of chiropractic patients [8], revealing limited use of chiropractors for rehabilitation to optimise function and meet the needs of people with disabilities. Trends also display decreased utilisation across ethnic minorities and culturally diverse populations, possibly due to a lack of familiarity with the profession, differing healthcare beliefs, and varying cultural backgrounds that shape views on disability and the delivery and uptake of care [56, 57]. For example, in some cultures, some may view disability as a medical issue, while other cultures may see it as part of a social belief, particularly where there may be a lack of willingness to engage in certain treatments or therapies, which can be influenced by how individuals perceive the cause of disability [56]. Another example is the role of family and caregiving in rehabilitation, which varies widely across cultures: some cultures place a strong emphasis on family support and involvement, whilst others focus on privacy [56]. We believe chiropractors already strengthen the delivery of rehabilitation through promoting equity, respect for diversity, and inclusivity which is foundational to chiropractic practice [58]. Chiropractic is under-utilised among people with disability and culturally diverse populations [59, 60], and therefore efforts should be taken by the profession to support participation for all who need it, across social contexts, and to increase demand for chiropractic care.
3. *Increased cultural responsiveness through improved cultural competency training and education.* Chiropractors can contribute to the strengthening of rehabilitation systems as they meet complex needs of patients with culturally responsive practice. In a cross-sectional survey of Canadian chiropractors, increased cultural competence was positively associated with Equality, Diversity, and Inclusion (EDI) training [61]. Where the cost of services and language were identified as barriers to providing chiropractic care, these results may inform the development of profession-specific training in cultural competence [61]. To ensure maximum impact, strategies that address diversity require chiropractors to commit to cultural competence training, thus enabling them to understand and respect the diverse beliefs and practices of their patients; to adopt and implement feedback mechanisms that address issues that arise in their geographical setting; to use generational interpreters or multilingual staff to overcome language barriers to ensure clear communication with patients; and curate care plans that are tailored to the individual's cultural background ensuring that their beliefs are considered. Furthermore, research has shown that culturally congruent care involves top-down and bottom-up approaches that integrate EDI practices at organisational and clinician levels [62]. Chiropractors adopting training and education opportunities that promote cultural competency may enable people to engage in life and reduce social isolation, strengthening their role to deliver rehabilitation in health systems.

### Challenges for the chiropractic profession

A recent systematic review by Newell et al., revealed persistent misconceptions about chiropractic’s scope of practice, scientific evidence base, and efficacy beyond spinal manipulation [63] which are possible determents of health systems to increase the uptake of chiropractor’s to deliver interventions for rehabilitation. Opportunities are furthered hindered despite chiropractic’s overlapping professional footprints with other health professions [36]. Here we present three challenges to chiropractic’s advanced integration in health systems to deliver rehabilitation, that include varying legislations within and between countries, limited recognition among other healthcare professionals, and improving multidisciplinary rehabilitation education.

1. *Varying access to, and regulation of chiropractic within and between countries.* Delivery of interventions for rehabilitation by chiropractors is often not covered under publicly funded health systems. High out-of-pocket costs for rehabilitation contribute to inequities in access to rehabilitation, especially in underserved or rural populations [64]. Chiropractic utilisation tends to occur primarily among people with higher income and higher levels of education [65], likewise, high-income countries have greater access to chiropractic [66]. Globally the rehabilitation workforce are fragmented and insufficient, attributable to the lack of common taxonomies, inadequate resources needed to monitor the workforce, and little or no political engagement or support [66]. From 193 United Nations member states, chiropractors are present in 90 countries, and overwhelming clustered in North America, Europe and Australia [66]. Inconsistent regulation and workforce recognition limits chiropractor’s role in delivering rehabilitation in health systems, particularly as regulation is largely absent in LMIC’s where rehabilitation need is greatest. Conversely, in high income countries, chiropractors are more likely to contribute to integrated, person-centred care focussed on optimising function and participation.
2. *Limited recognition of chiropractors, as providers of rehabilitation, among other healthcare professionals.* WHO has emphasised the development of strong multidisciplinary rehabilitation workforces as a priority [5]. Interventions commonly offered by chiropractors are consistent with clinical practice guidelines for low back pain, neck pain, whiplash, and headache [9–13], conditions that have high prevalence and high levels of associated disability. Despite a considerable body of literature and a growing evidence base, most health professionals have relatively low perceptions of chiropractors, which is in contrast to care-seeking patients, where up to 80–90% of patients with lumbar radiculopathy were “very satisfied” with care [67]. A recent example of the limited recognition of chiropractors amongst stakeholders is the Package of Interventions for Rehabilitation (PIR), Module 2: Musculoskeletal Conditions, a published WHO resource that outlines essential rehabilitation interventions for health conditions [68]. While this 2023 article focuses on interventions and not professions, and is designed to be profession-neutral, chiropractic is omitted. With 568 million people worldwide living with low back pain [69] and 2.4 billion people with problems that could benefit from rehabilitation [2], it is imperative that governments, stakeholders, and other healthcare professionals recognise it is relevant that chiropractors be integrated, and ultimately strengthen,the workforce to deliver rehabilitation in health systems.
3. *Improving students’ knowledge, skills and attitudes in relation to rehabilitation.* Rehabilitation is increasingly recognised as an essential component of chiropractic education, however it is currently unknown how rehabilitation is taught and how chiropractic students navigate multidisciplinary rehabilitation teams. A study involving chiropractic students in the United States revealed that students’ attitudes, beliefs, and work recommendations for those with chronic back pain were inconsistent with widely accepted multidisciplinary guidelines [70]. Integrating the ICF [3] into curricular may enhance students’ ability to identify functional barriers to recovery, and develop rehabilitation strategies that prioritise optimising function and participation. Gaps in chiropractic students’ and postgraduate learners’ rehabilitation knowledge, skills and attitudes exist, thus prompting the development of an initial set of proposed integrative health care competencies [71]. We believe, understanding and strengthening rehabilitation education through curricula aligned with the ICF framework will prepare future chiropractors to participate effectively in multidisciplinary rehabilitation teams to optimise patient-centred and functional outcomes.

### A call to action

Chiropractors are an important, yet often underutilised, contributor to the delivery of rehabilitation in health systems. A conceptual model of the qualities preferred in a chiropractor by key stakeholders in a neurorehabilitation setting [38] complement the chiropractors’ focus on restoring function and reducing pain through person-centred care. We urge healthcare leaders, policymakers, and chiropractors to recognise and support chiropractic’s role through implementation science, HPSR, and interprofessional education. Below, we make three “calls to action” that we believe will likely enhance patient outcomes, reduce healthcare costs, and promote the delivery of rehabilitation for people with health conditions throughout the life course, and across the continuum of care.

**Fig. 2:**
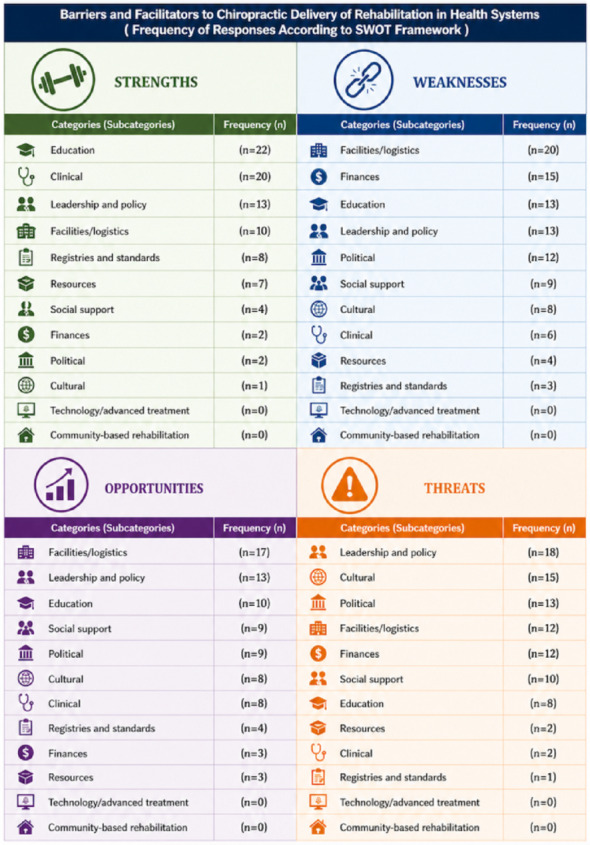
Frequencies of responses to predefined categories, subcategories, and barriers and facilitators to chiropractic delivery of rehabilitation in health systems, according to the SWOT framework

1. *Validate the efficacy, cost-effectiveness and efficiencies of the delivery of rehabilitation by chiropractors.* Theory driven implementation strategies, such as that by Roseen et al. [72], attempt to increase adoption of chiropractic, assessing feasibility, acceptability, fidelity and healthcare utilisation. The profession must overcome barriers that limit their adoption in public systems, primary care and hospital settings. Research that provides detailed information on different user groups, across service provision levels and for rehabilitation phases (acute, post-acute, and long-term) is desperately needed to provide evidence for knowledge gaps in the delivery of rehabilitation by chiropractors. The Guide for Rehabilitation Workforce Evaluation (GROWE) provides a method and tools for collecting, analysing, and interpreting key rehabilitation workforce data [73] and should be used in future research to identify chiropractic workforce gaps, training needs, and integration opportunities. Independently governed and sustainable chiropractic research infrastructure that aligns GROWE’s recommendations to produce workforce gap analyses, service delivery models and training credentials, is precisely what policy makers and rehabilitation planners need, and this data will strengthen the profession’s case for inclusion in national and global rehabilitation strategies. Directing membership levies from national associations and organisations such as the World Federation of Chiropractic, dedicated to validating chiropractic’s role in strengthening rehabilitation in health systems is immediately warranted.
2. *Advocate for policies that support widespread recognition and the inclusion of chiropractors in the delivery of rehabilitation.* Health policy and systems research is the study of how health policies impact collective health goals. It generates evidence to inform policymakers on how to design, implement, and improve the delivery of rehabilitation at a systems level. Where chiropractic has traditionally defended its professional sovereignty, a shift is needed so that health systems can recognise the function our workforce performs. Specifically, the narrow perception that chiropractors only do manual therapy, overlooks our delivery of interventions that fall within a coordinated, goal-directed process aimed at optimising function and participation. Efforts to undertake HPSR will strengthen chiropractic infrastructure, improve service quality and ensure equitable access to rehabilitation. It is also important that the profession disseminate evidence on cost-effectiveness [53], improved patient outcomes [74], collaborations with interdisciplinary teams [74], and participation in public health discussions [75, 76]. A recent World Federation of Chiropractic Congress workshop identified strategies to strengthen rehabilitation in local, regional and global health systems, to improve chiropractic utilisation and decrease the burden of conditions that require rehabilitation (Fig. 2). Further, professional efforts should include the immediate use of WHO and ICF taxonomies (function, participation, disability), working under rehabilitation governance structures and designing roles that enhance chiropractors in multidisciplinary rehabilitation teams [77, 78].
3. *Enhance interprofessional education and advance rehabilitation in chiropractic curricular.* There is a shift towards competency-based education in health professions education [79, 80]. While several frameworks are available [81], one such framework is the Interprofessional Education Collaborative (IPEC), which focuses on interprofessional collaboration for team-based care. Interprofessional educational initiatives show that chiropractic trainees are receptive to interprofessional education and believe interprofessional education enhances their ability to understand clinical problems and collaborate with other health-care professionals [82]. In real-world integration, interprofessional educational initiatives and integration of chiropractors into primary-care teams showed improvements in collaborative competency, better understanding and attitudes toward chiropractic, and acceptance of chiropractors working within funded primary care teams [40]. We believe improving knowledge of the scope and function of our workforce by patients and other health care practitioners may increase system-level access, improve acceptance into multidisciplinary rehabilitation teams and facilitate timely patient care. Conversely, increasing chiropractors’ knowledge and experience with collaborating with other health professions can enhance care efficiency, patient satisfaction, and clinical outcomes. When health care professionals are educated together, they can foster a collaborative approach to future patient care [83].

## Conclusion

We believe, chiropractors can play an important role in strengthening rehabilitation in health systems as they deliver interventions for rehabilitation for people with health conditions throughout the life course, and across the continuum of care. Through patient-centred care, chiropractors have contributed meaningfully in multidisciplinary rehabilitation teams, primary care, and hospital settings. However substantial barriers remain, including inconsistent regulation, variable access globally and poor interprofessional recognition. As a result, chiropractors remain underutilised to meet the rehabilitation needs of people worldwide. Our call for action seeks theory driven implementation strategies for chiropractors to be embedded within health systems to deliver rehabilitation, data on chiropractic workforce gaps and rehabilitation training needs, advocacy for improved recognition through HSPR and enhanced interprofessional education through advanced rehabilitation curricular. As a profession we must align with global rehabilitation priorities and uptake taxonomies to enable equitable access to chiropractors focused on optimising function and participation.

## Data Availability

Not applicable.

## Abbreviations

EDI: Equity, diversity and inclusion
GROWE: Guide for Rehabilitation Workforce Evaluation
HSPR: Health policy and systems research
IPEC: Interprofessional Education Collaborative
LMICs: Low- and middle-income countries
PIR: Package of Interventions for Rehabilitation
VA: Veterans Affairs
WHO: World Health Organization
